# 15-Deoxy-Δ^12,14^ Prostaglandin J_2_ Reduces the Formation of Atherosclerotic Lesions in Apolipoprotein E Knockout Mice

**DOI:** 10.1371/journal.pone.0025541

**Published:** 2011-10-07

**Authors:** Takahiro Seno, Masahide Hamaguchi, Eishi Ashihara, Masataka Kohno, Hidetaka Ishino, Aihiro Yamamoto, Masatoshi Kadoya, Kaoru Nakamura, Ken Murakami, Satoaki Matoba, Taira Maekawa, Yutaka Kawahito

**Affiliations:** 1 Department of Inflammation and Immunology, Graduate School of Medical Science, Kyoto Prefectural University of Medicine, Kyoto, Japan; 2 World Premier International Research Center, Immunology Frontier Research Center, Osaka University, Osaka, Japan; 3 Department of Molecular Cell Physiology, Graduate School of Medical Science, Kyoto Prefectural University of Medicine, Kyoto, Japan; 4 Department of Cardiovascular Medicine, Graduate School of Medical Science, Kyoto Prefectural University of Medicine, Kyoto, Japan; 5 Department of Transfusion Medicine and Cell Therapy, Kyoto University Hospital, Kyoto, Japan; University of Tor Vergata, Italy

## Abstract

**Aim:**

15-Deoxy-Δ^12,14^ Prostaglandin J_2_ (15d-PGJ_2_) is a ligand of peroxisome proliferator-activated receptor γ (PPARγ) having diverse effects such as the differentiation of adipocytes and atherosclerotic lesion formation. 15d-PGJ_2_ can also regulate the expression of inflammatory mediators on immune cells independent of PPARγ. We investigated the antiatherogenic effect of 15d-PGJ_2_.

**Methods:**

We fed apolipoprotein (apo) E-deficient female mice a Western-type diet from 8 to 16 wk of age and administered 1 mg/kg/day 15d-PGJ_2_ intraperitoneally. We measured atherosclerotic lesions at the aortic root, and examined the expression of macrophage and inflammatory atherosclerotic molecules by immunohistochemical and real-time PCR in the lesion.

**Results:**

Atherosclerotic lesion formation was reduced in apo E-null mice treated with 15d-PGJ_2_, as compared to in the controls. Immunohistochemical and real-time PCR analyses showed that the expression of MCP-1, TNF-α, and MMP-9 in atherosclerotic lesions was significantly decreased in 15d-PGJ_2_ treated mice. The 15d-PGJ_2_ also reduced the expression of macrophages and RelA mRNA in atherosclerotic lesions.

**Conclusion:**

This is the first report 15d-PGJ_2_, a natural PPARγ agonist, can improve atherosclerotic lesions in vivo. 15d-PGJ_2_ may be a beneficial therapeutic agent for atherosclerosis.

## Introduction

Atherosclerosis is now recognized as a chronic inflammatory condition and remains the major cause of cardiovascular disease [1]. Over the past two decades, data have emerged showing that immune cells, especially macrophages, are involved in the formation of atherosclerotic plaques.

Peroxisome proliferator-activated receptor γ (PPARγ) is a member of the nuclear receptor superfamily, and is expressed in arterial wall cells, such as vascular smooth muscle cells, and macrophages [2]. Thiazolidinediones (TZDs), which are some of the most common PPARγ ligands, are insulin-sensitizing antidiabetic agents causing the improvement of hypertension and hypertriglyceridemia, both of which represent major risk factors for atherosclerosis. TZDs can improve atherosclerosis by decreasing these risk factors. A previous study indicated that troglitazone, a TZD, had pleiotropic anti-atherosclerotic effects on the expression of CD36 in atherosclerotic lesions and the serum level of HDL, but the details of the mechanisms were not clear [3]. Another function of TZDs comprises its anti-mitogenic effect on vascular smooth muscle cells [4]. TZDs also inhibit macrophage activation [5], monocyte migration [6], inflammatory cytokine secretion by monocytes [7]–[9], and the expression of cell adhesion molecules expressed by vascular endothelial cells [10], [11]. Thus, a variety of anti-atherosclerotic effects of TZDs are associated with the regulation of inflammation caused by macrophages, but elucidation of the mechanisms in detail is required.

The J series of prostaglandins (PGs) have been demonstrated to regulate processes like inflammation and tumorgenesis [12]. 15-Deoxy-Δ^12,14^ Prostaglandin J_2_ (15d-PGJ_2_) is a metabolite of PGD_2_, and is produced by mast cells, T cells, platelets and alveolar macrophages. 15d-PGJ_2_ is recognized as an endogenous ligand for the intranuclear receptor PPARγ [13], which leads to inhibition of phorbol ester-induced nitric oxide and macrophage-derived cytokines, i.e., tumor necrosis factor-α (TNF-α), IL-1 and IL-6. 15d-PGJ_2_ inhibits gene expression in part by antagonizing the activities of transcription factors such as activator protein-1 and nuclear factor-κB (NF-κB) [7]. Furthermore, 15d-PGJ_2_ has an anti-atherosclerotic effect as a ligand of PPARγ. Previous studies have been shown that 15d-PGJ_2_ dose-dependently inhibits several functions of endothelial cells related to angiogenesis, such as proliferation, morphogenesis and migration in vitro [14]–[16]. Another study revealed that an increased plasma 15d-PGJ_2_ concentration was associated with the early and late neurological outcomes, and a smaller infarct volume in ischemic stroke patients [17]. However, it remains unknown whether or not 15d-PGJ_2_ has an anti-atherogenic effect in vivo. To investigate the effects of 15d-PGJ_2_ on atherosclerotic lesion formation, we treated apo E–knockout mice, an animal model of atherosclerosis, with 15d-PGJ_2_, and then examined the atherosclerotic lesions.

## Methods

### Animals

Apo E-knockout mice (C57BL/6J-Apoe^tm1Unc^) were purchased from the Jackson Laboratory (B6 background; The Jackson Laboratory, Bar Habor, ME) [18]. These mice were produced by backcrossing the Apoe^tm1Unc^ mutation 10 times to C57BL/6J mice. Mice were maintained under specific pathogen-free conditions, and allowed ad libitum access to food and water. Thirty female animals aged 8 wk (15 as controls and 15 for the 15d-PGJ_2_ experiments) were fed the Western-type diet containing 0.2% cholesterol and 21% saturated fat (Oriental Yeast, Tokyo, Japan) for 8 wk. All mice received intraperitoneal injections of (1) PBS (control group), and (2) 15d-PGJ_2_ (Cayman Chemicals, Ann Arbor, USA), 1 mg/kg/day (15d-PGJ_2_ group), for 8 wk with a high fat diet. Administration route and dosage of 15d-PGJ_2_ were based on our previous study [19]. The animal care and experimental procedures conformed to the regulations of the Animal Research Committee, Graduate School of Medicine, Kyoto University.

### Quantitative analyses of atherosclerotic lesions

Following blood collection, mice aged 16 wk treated with PBS or 15d-PGJ_2_ were examined. After overnight fasting, blood was collected from the cardiac cavity and analyzed for the lipid profile. Also, aortae from the ascending portion to the end of the thoracic aorta were removed and washed meticulously in cold PBS to remove attached hematocytes and tissue fragments on the outside the aortae. Proximal aortic roots were used for quantitative analysis of the atherosclerotic area and whole thoracic aortae for real-time polymerase chain reaction (PCR) analysis.

Atherosclerotic lesions were quantitatively analyzed as previously described [20], [21]. In brief, the basal portion of the heart and proximal aortic root were excised, embedded in OCT compound (Sakura Finetek, Tokyo, Japan), and then frozen in liquid nitrogen. Three serial cryosections per one aortic root of 10 µm thickness, at 40 µm intervals, of the aortic sinus were stained with oil-red O (Wako Pure Chemical Industries Ltd, Osaka, Japan) and hematoxylin. Other three cryosections per one aortic root were stained with Masson's trichrome (Kyodo Byori, Kobe, Japan) for cellular components (red) and fibrous tissue (blue). Lesion images were captured with a DMBA210 microscope (Shimadzu Rika, Tokyo, Japan) equipped with Motic Images Plus2.2s software (Shimadzu Rika, Tokyo, Japan). The captured images were analyzed with Image J software (NIH, USA). We calculated the oil-red O positive area, fibrotic area and aortic root area and compared the average data of three sections. A blind observer analyzed the lesions.

### Immunohistochemistry

Immunohistochemistry was performed on 10 µm thick cryosections as described above. Tissue sections were immersed for 30 min in 0.3% hydrogen peroxide in methanol to block endogenous peroxidase activity. Nonspecific binding sites were saturated by exposure to 0.2% bovine serum albumin and normal serum for 30 min. Rat monoclonal anti-mouse macrophages (MOMA-2; AbD Serotec, Oxford, United Kingdom), goat anti-mouse monocyte chemoattractant protein-1 (MCP-1; Santa Cruz Biotechnology Inc., California, USA), rabbit anti-mouse macrophage migration inflammatory factor (MIF; Life Technologies, California, USA), goat anti-mouse TNF-α (R&D Systems, Minnesota, USA), goat anti-mouse matrix metallopeptidase-9 (MMP-9; Santa Cruz Biotechnology Inc., California, USA) and goat anti-mouse PPARγ (Santa Cruz Biotechnology Inc., California, USA) Abs were used as primary Abs. These primary anti-mouse macrophage Abs (1/50 dilution in PBS), anti-mouse MCP-1 Abs (1/100 dilution in PBS), anti-mouse MIF Abs (1/00 dilution in PBS), anti-mouse TNF-α Abs (1/100 dilution in PBS), anti-mouse MMP-9 Abs (1/100 dilution in PBS), anti-mouse PPARγ Abs (1/100 dilution in PBS) and control normal serum were applied to tissue sections, followed by incubation overnight at 4°C. The slides were treated with 0.2% glutaraldehyde. Then biotinylated secondary Abs and streptavidin–horseradish peroxidase were used for detection (Nichirei Bioscience, Tokyo, Japan) for 30 min. Signals were developed with a DAB Peroxidase Substrate Kit, 3,3′-diaminobenzidine (Vector Laboratories, Burlingame, USA). Positive staining was indicated by brownish black deposits, and counterstaining was performed with hematoxylin. The images were captured with a DMBA210 microscope, and the captured images were analyzed with Image J software (NIH, USA), the ratios of the positive area to the whole cross-sectional aortic wall area being calculated. Each data was average of three sections. A blind observer analyzed the lesions.

### Real-time reverse-transcription polymerase chain reaction

Several gene expressions such as MCP-1, MIF, TNF-α, MMP-9 and RelA (p65), were analyzed by real-time quantitative RT-PCR using the TaqMan system based on real-time detection of accumulated fluorescence. Total RNA was extracted from whole thoracic aortae by homogenization in an RNeasy Fibrous Tissue Mini Kit (Qiagen Japan, Tokyo, Japan). cDNA was synthesized by reverse transcription with a Clontech Advantage RT-for-PCR Kit (Takara Bio Inc., Otsu, Japan). Quantitative real-time reverse-transcription polymerase chain reaction was performed using an Applied Biosystems 7300 Real-Time PCR System (Applied Biosystems, California, USA), followed by analysis involving software detection system (SDS version 1.9) software. Gene expression was normalized as to 18S rRNA (Applied Biosystems).

### Lipid metabolism

After overnight fasting, blood was collected from the cardiac cavity of mice aged 16 wk and analyzed for the lipid profile. The plasma chylomicron (CM), very low density lipoprotein (VLDL), low density lipoprotein (LDL), and high density lipoprotein (HDL) levels were determined by use of a high-sensitivity lipoprotein-profiling system by high-performance liquid chromatography (HPLC) (Skylight Biotech, Inc., Akita, Japan) [22]. HPLC with gel permeation columns was performed to classify and quantify lipoproteins on the basis of differences in particle size [23].

### Statistical analysis

The results were expressed as means ± SE and analyzed by means of Student's t test (GraphPad Prism 5.03; Graph Pad Software Inc., CA, USA). Values of *p*<0.05 were considered statistically significant.

## Results

### Body weight

**Figure 1:**
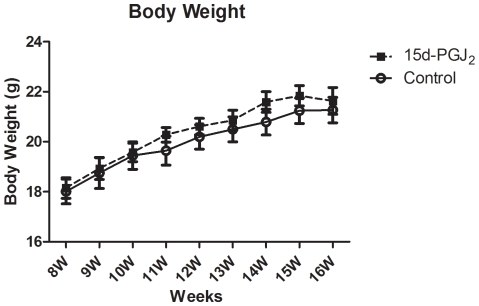
Body weights of apo E-knockout mice treated with PBS or 15d-PGJ_2_ from 8 to 16 weeks of age. From the 8th week to 16th week, female mice were randomized to receive a Western-type diet and PBS or 1 mg/kg/day of 15d-PGJ_2_ (n = 15 animals for each group). At 16th week, body weight of 15d-PGJ2 treated mice tended to be higher than controls, but it was not significantly different (21.6±4.2 g and 21.2±3.9 g, respectively, *p* = 0.6). Statistical analyses were performed with Student's t test.

From the 8th week to 16th week, mice were randomized to receive a Western-type diet and PBS or 15d-PGJ_2_. Figure 1 shows the change of body weight for observation period. At 16th week, body weight of 15d-PGJ_2_ treated mice tended to be higher than controls, but it was not significantly different (21.6±4.2 g and 21.2±3.9 g, respectively, *p* = 0.6). Body weight did not decrease after intraperitoneal administration of PBS or 15d-PGJ_2_.

### Atherosclerotic lesions in the aortic sinus

**Figure 2:**
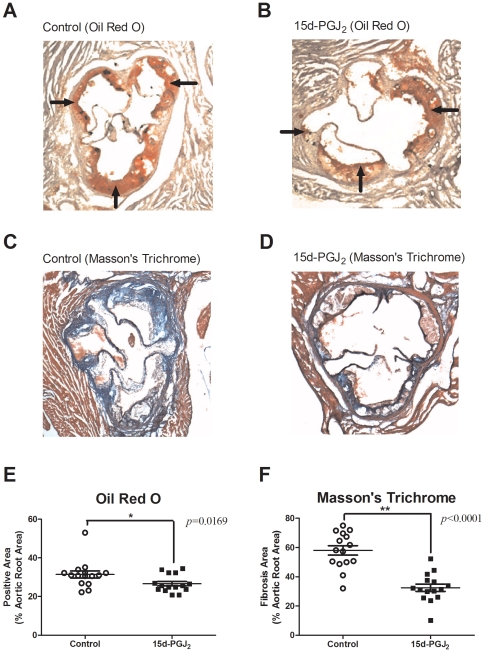
Representative oil red O stained sections and Masson's trichrome stained sections from the proximal aortae of apo E-knockout mice, and prevalence of atherosclerotic lesions. A and B are representative oil red O stained sections from the proximal aortae of apo E-knockout mice. C and D are representative Masson's trichrome stained sections. Apo E knockout-mice were fed a Western-type diet and treated with PBS (control group) (n = 15) (A, C) or 1 mg/kg/day 15d-PGJ_2_ (15d-PGJ_2_ group) (n = 15) (B, D) for 2 mo. Cross-sections of proximal aortae were stained with oil red O and counterstained with hematoxylin. Black arrows indicate the positive lesions. We plotted the prevalence of oil red O positive areas in cross-sections of whole atherosclerotic lesions in each group (E). Short lines indicate the means ± SD. The prevalence in controls were 31.44±1.811% and 26.63±1.169%, respectively. Cross-sections of proximal aortae were also stained with Masson's trichrome for cellular components (smooth muscle cells: pink, and red blood cells: red) and fibrous tissue (blue). We plotted the prevalence of fibrosis areas in cross-sections of whole atherosclerotic lesions in each group (F). Short lines indicate the means ± SD. The prevalence of fibrotic areas in controls were 58.05±3.218% and 32.48±2.535%, respectively. Statistical analyses were performed with Student's t test. *p<0.05, **p<0.01.

To determine the factors mediating the anti-atherosclerotic effect of 15d-PGJ_2_, we compared the area of oil red O-positive lesions and fibrotic lesions in cross-sections of the aortic wall between the control and 15d-PGJ_2_ groups (n = 15, respectively). Representative micrographs are presented in Figure 2. Typical atheromas with well-developed, lipid-rich cores and foam cell infiltration were observed. The prevalence of oil red O positive areas in cross-sections of whole atherosclerotic lesions were 31.44±1.811% in the controls and 26.63±1.169% in the 15d-PGJ_2_ groups. The prevalence of Masson's trichrome stained fibrotic areas were 58.05±3.218% and 32.48±2.535%, respectively.

### Immunohistochemistry of the atherosclerotic lesions

**Figure 3:**
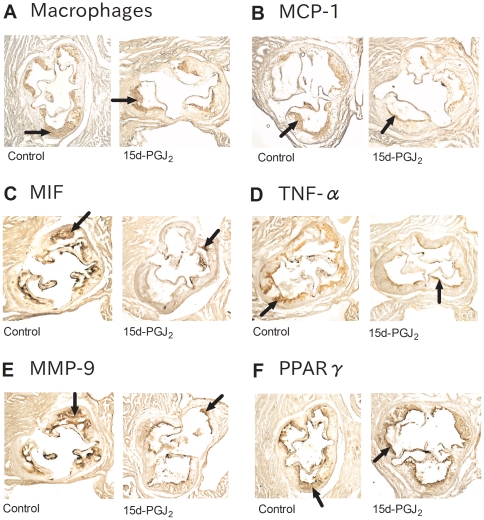
Representative sections with immunohistochemical analysis. Apo E-knockout mice were fed a Western-type diet and treated with PBS (control group) (n = 10) or 1 mg/kg/day 15d-PGJ_2_ (15d-PGJ_2_ group) (n = 10) for 2 mo. Representative cross-sections of the aortic sinus were stained with MOMA-2 (A), which detected macrophages, and MCP-1 Abs (B), MIF Abs (C), TNF-α Abs (D), MMP-9 Abs (E), PPARγ Abs (F), and counterstained with hematoxylin. Right sections are control group and left ones are 15d-PGJ_2_ group in each figure. Black arrows indicate the positive lesions.

**Figure 4:**
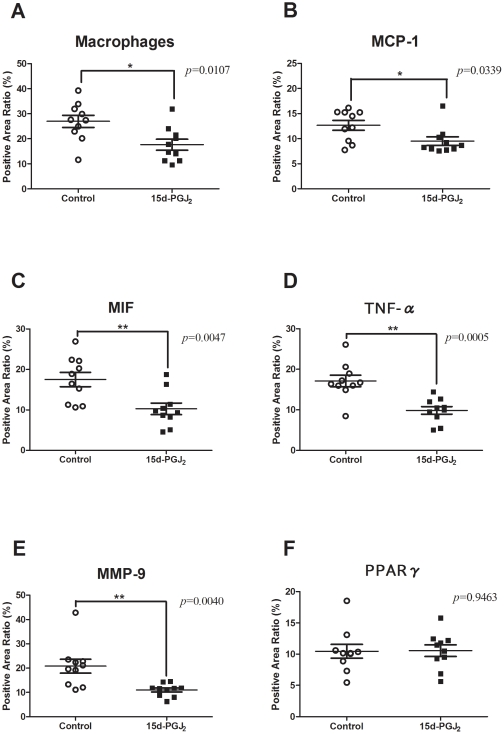
Prevalence of atherosclerotic lesions with immunohistochemical analysis. Apo E-knockout mice were fed a Western-type diet and treated with PBS (control group) (n = 10) or 1 mg/kg/day 15d-PGJ_2_ (15d-PGJ_2_ group) (n = 10) for 2 mo. Representative cross-sections of the aortic sinus were stained with MOMA-2, which detected macrophages, and MCP-1 Abs, MIF Abs, TNF-α Abs, MMP-9 Abs, PPARγ Abs, and counterstained with hematoxylin. We plotted the prevalence of positive areas in cross-sections of whole atherosclerotic lesions in each group. Short lines indicate the means ± SD. The prevalence of macrophage (A), immunoreactive MCP-1 (B), MIF (C), TNF-α (D) and MMP-9 (E) in atherosclerotic lesions of apo E-knockout mice treated with PBS or 15d-PGJ2 was examined. The prevalence of macrophage in the control and 15d-PGJ_2_ groups were 26.97±2.437% and 17.64±2.194%, respectively. The prevalence of immunoreactive MCP-1 (9.508±0.8518% vs 12.65±0.9788%, *p* = 0.0339), MIF (10.28±1.402% vs 17.53±1.762%, *p* = 0.0047), TNF-α (9.853±0.9462% vs 17.12±1.412%, *p* = 0.0005) and MMP-9 (11.02±0.8208% vs 20.80±2.846%, *p* = 0.0040) were decreased in the 15d-PGJ_2_ groups. But the prevalence of PPARγ (F) was not different between both groups (10.55±0.9217% vs 10.46±1.104%, *p* = 0.9463). *p<0.05, **p<0.01, with Student's *t* test.

We explored the mechanism underlying the anti-atherosclerotic effect of 15d-PGJ_2_. Immunohistochemistry was performed with MOMA-2, which detected macrophages, anti-MCP-1 Abs, anti- MIF Abs, anti- TNF-α Abs, and anti-MMP-9 Abs. We compared the prevalence of positive areas in the aortic root between the control and 15d-PGJ_2_ groups (n = 10, respectively). The 15d-PGJ_2_ group exhibited significant lower expression of MCP-1 (9.508±0.8518% vs 12.65±0.9788%, *p* = 0.0339), MIF (10.28±1.402% vs 17.53±1.762%, *p* = 0.0047), TNF-α (9.853±0.9462% vs 17.12±1.412%, *p* = 0.0005), MMP-9 (11.02±0.8208% vs 20.80±2.846%, *p* = 0.0040) and macrophages (17.64±2.194% vs 26.97±2.437%, *p* = 0.0107), compared with in control group (Figure 3A–3E, Figure 4A–4E). But the prevalence of PPARγ was not different between both groups (10.55±0.9217% vs 10.46±1.104%, *p* = 0.9463) (Figure 3F, Figure 4F).

### Gene expressions in the thoracic aorta

**Figure 5:**
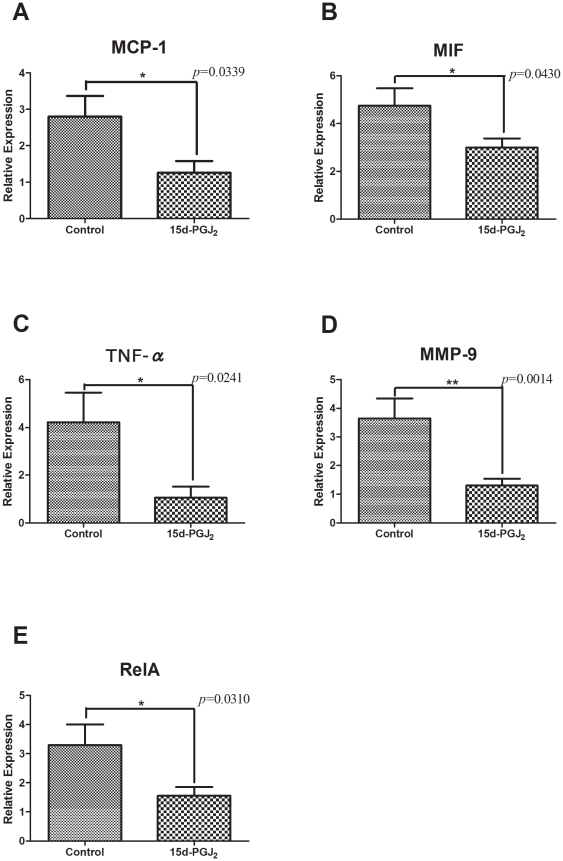
Comparison of gene expressions in the thoracic aorta between the control and 15d-PGJ_2_ treated groups by real-time PCR analysis. Apo E-knockout mice were fed a Western-type diet and treated with PBS (control group) or 1 mg/kg/day 15d-PGJ_2_ (15d-PGJ_2_ group) for 2 mo. Thoracic aortae were removed and total RNA was extracted from them. cDNA was synthesized by reverse transcription, and quantitative real-time PCR was performed. The relative gene expression values were calculated. The relative expressions were significantly decreased in the 15d-PGJ_2_ group, MCP-1 (1.263±0.3193 vs 2.802±0.5627, *p* = 0.0339) (A), MIF (2.985±0.3860 vs 4.745±0.7347, *p* = 0.0430) (B), TNF-α (1.059±0.4625 vs 4.220±1.236, *p* = 0.0241) (C), MMP-9 (1.304±0.2344 vs 3.644±0.6947, *p* = 0.0014) (D) and RelA (1.551±0.2995 vs 3.294±0.7093, *p* = 0.0310) (E), compared with in the control group. *p<0.05, **p<0.01, with Student's *t* test.

Figure 5 shows the results of quantitative real-time PCR analysis of MCP-1, MIF, TNFα, MMP-9 and RelA gene expressions in thoracic aortae. All of those gene expressions were significantly decreased in the 15d-PGJ_2_ group (n = 10, respectively), MCP-1 (1.263±0.3193 vs 2.802±0.5627, *p* = 0.0339), MIF (2.985±0.3860 vs 4.745±0.7347, *p* = 0.0430), TNF-α (1.059±0.4625 vs 4.220±1.236, *p* = 0.0241), MMP-9 (1.304±0.2344 vs 3.644±0.6947, *p* = 0.0014) and RelA (1.551±0.2995 vs 3.294±0.7093, *p* = 0.0310), compared with in the control group. 15d-PGJ_2_ also reduced the expressions of these atherosclerotic markers at the gene level. It indicated that 15d-PGJ_2_ led to downregulation of these gene expressions via NF-κB, and these results were almost comparable with immunohistochemistry. In addition, ligand-induced negative-feedback was not identified in our study.

### 15d-PGJ_2_ treatment improves the lipid profile

**Figure 6:**
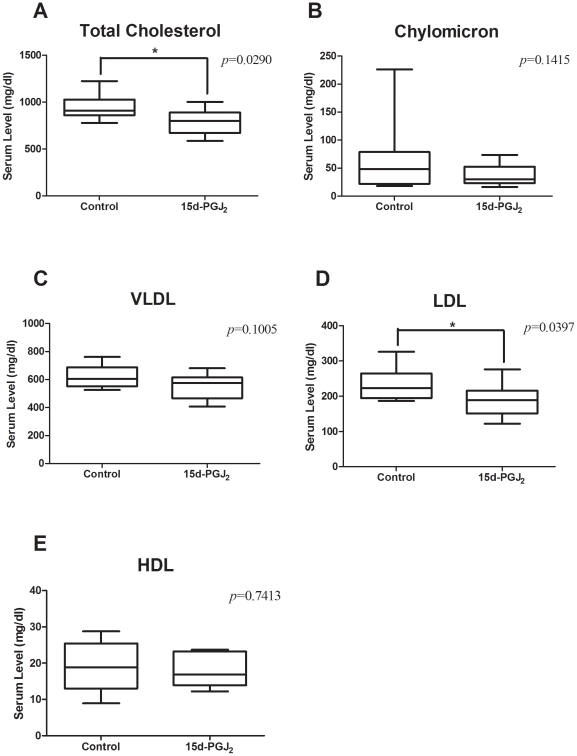
Serum lipid levels in the controls and the 15d-PGJ_2_ group. Blood was collected from the cardiac cavity of mice aged 16 wk and analyzed for the lipid profile. The plasma chylomicron (CM) (B), very low density lipoprotein (VLDL) (C), low density lipoprotein (LDL) (D), and high density lipoprotein (HDL) (E) levels were determined by use of a high-sensitivity lipoprotein-profiling system by high-performance liquid chromatography. The total serum cholesterol level (A) was significantly lower in the15d-PGJ_2_ group than in the control group (795.5±39.31 mg/dl vs 944.1±49.04 mg/dl, *p* = 0.029). Especially LDL was significantly reduced in the 15d-PGJ_2_ group (186.9±13.49 mg/dl vs 234.3±16.60 mg/dl, *p* = 0.0397). CM, VLDL and HDL were not different between the control and 15d-PGJ_2_ groups, 36.96±4.999 mg/dl vs 68.13±23.98 mg/dl, 553.5±26.67 mg/dl vs 622.7±28.02 mg/dl, 18.14±1.264 mg/dl vs 19.01±2.562 mg/dl, respectively. *p<0.05, with Student's *t* test.

We performed analyses of lipid levels at the end of this study. Pooled plasma from all mice was subjected to HPLC. Lipoproteins were separated in CM, VLDL, LDL, and HDL. The total serum cholesterol level was significantly lower in the15d-PGJ_2_ group than in the control group (795.5±39.31 mg/dl vs 944.1±49.04 mg/dl, *p* = 0.029) (Figure 6A). Especially LDL was significantly reduced in the 15d-PGJ_2_ group (186.9±13.49 mg/dl vs 234.3±16.60 mg/dl, *p* = 0.0397) (Figure 6D). CM and VLDL tended to be lower than in controls, but the difference was not significant (36.96±4.999 mg/dl vs 68.13±23.98 mg/dl, p = 0.1415; 553.5±26.67 mg/dl vs 622.7±28.02 mg/dl, *p* = 0.1005, respectively) (Figure 6B and C). The HDL level was not different between the control and 15d-PGJ_2_ groups (18.14±1.264 mg/dl vs 19.01±2.562 mg/dl, *p* =  0.7413) (Figure 6E).

## Discussion

15d-PGJ_2_ is a ligand of PPARγ, which acts to atherosclerosis formation. In this study, we fed apo E-deficient mice a Western-type diet and administered 15d-PGJ_2_. We measured the cross-sectional atherosclerotic area in the proximal aorta and examined the expression of several atherosclerotic markers in the lesions. The atherosclerotic area, represented by lipid accumulation and fibrous tissue, significantly decreased in apo E-null mice treated with 15d-PGJ_2_. Immunohistochemical and real-time PCR analyses showed that the expressions of MCP-1, MIF, TNF-α and MMP-9 in atherosclerotic lesions were significantly decreased. The 15d-PGJ_2_ also reduced the expression of RelA mRNA in atherosclerotic lesions. This study suggests that 15d-PGJ_2_ has an anti-atherosclerotic effect.

Atherosclerosis is an inflammatory disease. The lesions in atherosclerosis represent a series of highly specific cellular and molecular responses that can best be described, overall, as an inflammatory disease [24]. Atherosclerosis formation consists of several steps. The earliest changes that precede the formation of lesions of atherosclerosis take place in the endothelium. These changes include migration of leukocytes into the artery wall, which is mediated by MCP-1 [24]. Fatty streaks initially consist of lipid-laden monocytes and macrophages together with T lymphocytes. Later they are joined by various numbers of smooth-muscle cells. The steps involved in this process include T cell activation, foam-cell formation, which is mediated by TNF-α. As the advanced change, thinning of the fibrous cap is apparently due to the continuing influx and activation of macrophages, which release metalloproteinases such as MMP-9, and other proteolytic enzymes at these sites. MIF affects cell proliferation in lesions and elastolytic/collagenolytic cysteine protease expression. MIF may act as do other cytokines (eg, TNF-α) to enhance protease expression and vascular cell proliferation, processes that occur during atherogenesis [25]. Our data showed that 15d-PGJ_2_ inhibited MCP-1, MIF, TNF-α and MMP-9 as indicated by real-time PCR as well as immunohistochemical analysis. A previous study showed that thiazolidinediones and 15d-PGJ_2_ inhibit macrophage proliferation in a dose-dependent manner, and significantly reduce the migration of monocytes induced by MCP-1 in vitro [26]. Also, MCP-1 is one of the important mediators at early change of atherosclerosis formation. On possibility is that 15d-PGJ_2_ act on various steps of atherosclerosis formation. Another possibility is that 15d-PGJ_2_ act on the early step of atherosclerosis formation, as a consequence, 15d-PGJ_2_ decrease the mediators at following steps.

15d-PGJ_2_ is one of the PPARγ-ligands [13] emerging as a key anti-inflammatory mediator via NF-κB inhibition, may play a role in the pathogenesis of atherosclerosis [2]. NF-κB family consists of RelA (p65), c-Rel, and RelB, as well as p105 and p100 and their processed forms, p50 and p52, respectively. NF-κB primarily exists as a p50/p65 heterodimer [27]. In our data, PPARγ expressed in atherosclerotic lesions in both controls and 15d-PGJ_2_ groups. In addition, the expression of RelA was decreased in 15d-PGJ_2_ groups. It is generally known that high dose of ligands lead to downregulation of its receptor expressions. Although it has not been reported about PPARγ agonist, a previous study revealed that treatment with GW1929, a selective PPARγ antagonist, enhanced PPARγ mRNA expressions in kidneys from hypertension model rats [28]. This study shows the possibility that high dose PPARγ also downregulate its receptor expression. But PPARγ expression was not changed in our results. It indicated that 15d-PGJ_2_ did not induce the negative-feedback in our study. On the other hand, the reduction of RelA was owing to NF-κB inhibition. 15d-PGJ_2_ induces some PPARγ-independent biological actions, such as inhibition of NF-κB signaling through covalent modifications of critical cysteine residues in IκB kinase and the DNA-binding domains of NF-κB subunits [29]. We presumed that 15d-PGJ_2_ inhibited NF-κB not only as a PPARγ agonist but also as PPARγ-independent actions.

High plasma concentrations of cholesterol, in particular those of LDL cholesterol, are one of the principal risk factors for atherosclerosis [24]. In this study, 15d-PGJ_2_ decreased serum total cholesterol level and LDL cholesterol level. This shows that 15d-PGJ_2_ reduces the principal risk factor of atherosclerosis. But the detail mechanisms remain an open question. Further studies are needed to elucidate this matter.

In conclusion, this is the first study demonstrating an anti-atherosclerotic effect of 15d-PGJ_2_ in vivo, using a rodent model. The mechanism of its effect remains to be elucidated in detail. However, our data indicate that 15d-PGJ_2_ exhibits ability as to an anti-atherosclerotic effect. These findings suggest that 15d-PGJ_2_ is a beneficial therapeutic reagent for both atherosclerosis.
